# Cell proliferation within small intestinal crypts is the principal driving force for cell migration on villi

**DOI:** 10.1096/fj.201601002

**Published:** 2016-10-20

**Authors:** Aimee Parker, Oliver J. Maclaren, Alexander G. Fletcher, Daniele Muraro, Peter A. Kreuzaler, Helen M. Byrne, Philip K. Maini, Alastair J. M. Watson, Carmen Pin

**Affiliations:** * Gut Health and Food Safety Research Programme, Institute of Food Research, Norwich, United Kingdom;; † Wolfson Centre for Mathematical Biology, Mathematical Institute, University of Oxford, Oxford, United Kingdom;; ‡ School of Mathematics and Statistics, University of Sheffield, Sheffield, United Kingdom;; § Bateson Centre, University of Sheffield, Sheffield, United Kingdom;; ¶ Department of Biochemistry, University of Cambridge, United Kingdom; and; ‖ Norwich Medical School, University of East Anglia, Norwich, United Kingdom

**Keywords:** mathematical model, epithelium, small intestine

## Abstract

The functional integrity of the intestinal epithelial barrier relies on tight coordination of cell proliferation and migration, with failure to regulate these processes resulting in disease. It is not known whether cell proliferation is sufficient to drive epithelial cell migration during homoeostatic turnover of the epithelium. Nor is it known precisely how villus cell migration is affected when proliferation is perturbed. Some reports suggest that proliferation and migration may not be related while other studies support a direct relationship. We used established cell-tracking methods based on thymine analog cell labeling and developed tailored mathematical models to quantify cell proliferation and migration under normal conditions and when proliferation is reduced and when it is temporarily halted. We found that epithelial cell migration velocities along the villi are coupled to cell proliferation rates within the crypts in all conditions. Furthermore, halting and resuming proliferation results in the synchronized response of cell migration on the villi. We conclude that cell proliferation within the crypt is the primary force that drives cell migration along the villus. This methodology can be applied to interrogate intestinal epithelial dynamics and characterize situations in which processes involved in cell turnover become uncoupled, including pharmacological treatments and disease models.—Parker, A., Maclaren, O. J., Fletcher, A. G., Muraro, D., Kreuzaler, P. A., Byrne, H. M., Maini, P. K., Watson, A. J. M., Pin, C. Cell proliferation within small intestinal crypts is the principal driving force for cell migration on villi.

The epithelial lining of the intestine undergoes continual renewal; adult stem cells at the base of intestinal crypts proliferate and differentiate into multiple functionally distinct epithelial subtypes, which then migrate upward to the villus tips to eventually be shed into the gut lumen (1, 2). Maintenance of the functional integrity of the intestinal epithelial barrier therefore requires tight coordination of these processes, with failure to regulate epithelial cell turnover resulting in cells’ escaping normal growth controls, development of inflammatory disease, and tumor formation.

In part because of current limitations impeding *in vivo* imaging of entire crypt–villus units over prolonged periods, it is still not clear exactly how these processes are interrelated. Passive mitotic pressure generated by cell division in the intestinal crypts, and subsequent gradual expansion in cell diameter along the crypt–villus axis, provides a plausible explanation for the steady continuous migration of epithelial cells (3, 4). Indeed, previous computational models suggest that these forces alone are sufficient to explain observed rates of cell migration, at least within the crypt (5–10). Conversely, other studies have reported continued epithelial cell migration or evidence for villus-to-crypt feedback in regulating proliferation rates when crypts were targeted with irradiation, ischemia, or cytotoxic agents (11–18). In addition, cell migration on the villus has been found to exhibit a circadian rhythm, which is not observed in cell proliferation in the crypt (19). Active migration processes, such as those seen during wound healing (20–23), have been proposed to explain apparent disparities between proliferation and migration rates, whereas an alternative explanation for uncoupling between crypt and villus cell migration is the contribution of whole villus contraction and expansion (24, 25).

The purpose of this work was to investigate whether cell proliferation within crypts is sufficient to explain the observed cell migration on villi, both during homeostasis and under altered conditions in which crypt cell proliferation is either reduced or temporarily inhibited. To this end, we used the thymine analogs 5-bromo-2′-deoxyuridine (BrdU) and 5-iodo-2′-deoxyuridine (IdU), for tracking proliferative cells and their descendants along the crypt–villus axis. BrdU, IdU, and similar thymine analogs are incorporated into newly synthesized DNA of dividing cells during the *S* phase (26, 27). The incorporated molecule is transmitted to daughter cells, regardless of whether they proliferate. If the exogenous administration of these molecules is discontinued, the cell label content is diluted by each cell division and is no longer detected after 4–5 generations (28). To quantify cell proliferation and migration, we have developed mathematical models to describe the temporal dynamics of labeled cells across the crypt–villus axis.

Applying this methodology, we studied the relationship between crypt cell production and villus cell migration in the proximal and distal small intestine of C57BL/6 mice; in transgenic Omomyc mice, which exhibit reduced cell proliferation in the intestinal epithelium (29); and in C57BL/6 mice treated with the cytostatic/cytotoxic agent cytosine arabinoside (Ara-C) at doses that temporarily halted cell proliferation.

## MATERIALS AND METHODS

### Animals

All animal experiments were conducted in accordance with the Home Office Animals (Scientific Procedures) Act 1986.

Female C57BL/6 mice, aged 8–12 wk, were supplied by Charles River (Margate, United Kingdom) and maintained at the University of East Anglia, United Kingdom. Male and female *TRE-Omomyc;actin-rtTA*, mice, aged 8–13 wk, were kindly provided by Prof. Gerard Evans (Gurdon Institute, University of Cambridge, United Kingdom). To induce omomyc expression, we treated *TRE-Omomyc;actin-rtTA* mice with doxycycline delivered in the drinking water (2 mg/ml), commencing 1 wk before the start of BrdU labeling.

### Proliferative cell labeling and tissue processing

The thymine analogs BrdU and IdU (both from Sigma-Aldrich, Paisley, United Kingdom) were administered at 50 mg/kg body weight by intraperitoneal injection. Time of day for delivery was consistent across experiments, to reduce any possible variation caused by proliferative circadian rhythms (30). At appropriate time points thereafter, mice were euthanized, and intestinal tracts were removed, flushed, dissected, and embedded and frozen in optimal cutting temperature medium or fixed for 24 h in 10% neutral-buffered formalin. Formalin-fixed tissues were then processed through a xylene/alcohol series and embedded in paraffin, and transverse sections of duodenum and ileum were prepared at 5 μm.

### Blocking proliferation using Ara-C

Mice were given an initial intraperitoneal injection of IdU 17 h before a single injection of Ara-C (Sigma-Aldrich) at 250 mg/kg body weight. Tissues were collected between 1 and 57 h thereafter, with BrdU administration 1 h before they were euthanized, at each time point.

### Immunohistochemistry and immunofluorescence

Intestinal sections were deparaffinized before blocking endogenous peroxidases by incubation in 1% H_2_O_2_ in methanol, and heat-induced antigen retrieval in 10 mM citric acid buffer (pH 6). BrdU- and IdU-labeled cells were detected with sheep anti-BrdU-biotin or mouse anti-IdU (AbCam, Cambridge, United Kingdom) followed by NeutrAvidin-horseradish peroxidase (HRP; Thermo Fisher Scientific, Loughborough, United Kingdom) or goat-anti-mouse-HRP (Dako, Glostrup, Denmark) detected using 3,3′-diaminobenzidine (DAB; Dako) and counterstained with hematoxylin. Mitoses were detected with rabbit monoclonal Ab (EPR17246) to histone 3 (H3) (phospho-S10; AbCam). Expression of omomyc in intestinal crypts and villi was detected, using rabbit anti-omomyc antibody, goat anti-rabbit-HRP secondary, and DAB detection, as above. Intestinal cryosections prepared at 5 μm were fixed in acetone/methanol before incubation with primary antibodies to BrdU (BU1/75-DyLight 550; Novus Biologicals, Abdingdon, United Kingdom) and/or against IdU (mouse anti-IdU clone 32D8.D9; AbCam) followed by goat-anti-mouse Alexa 488 (Thermo Fisher Scientific).

### Image acquisition and analysis

Images were acquired with a DMI-3000-B inverted microscope and DFC-310-FX digital camera (Leica, Wetzlar, Germany), with analysis performed in ImageJ software (National Institutes of Health, Bethesda, MD, USA) (31).

### Mathematical modeling and statistical analysis

#### A 2-compartment model of the temporal cell dynamics of BrdU pulse-labeled cells in the crypt–villus epithelial unit 

We developed a time-dependent mathematical model of the crypt–villus epithelial unit (CVEU) that distinguishes 2 physical compartments: the crypt compartment, which includes all proliferative cells in the CVEU and some nonproliferative cells, and the villus compartment, which contains all remaining nonproliferative cells.

**Figure 1.**
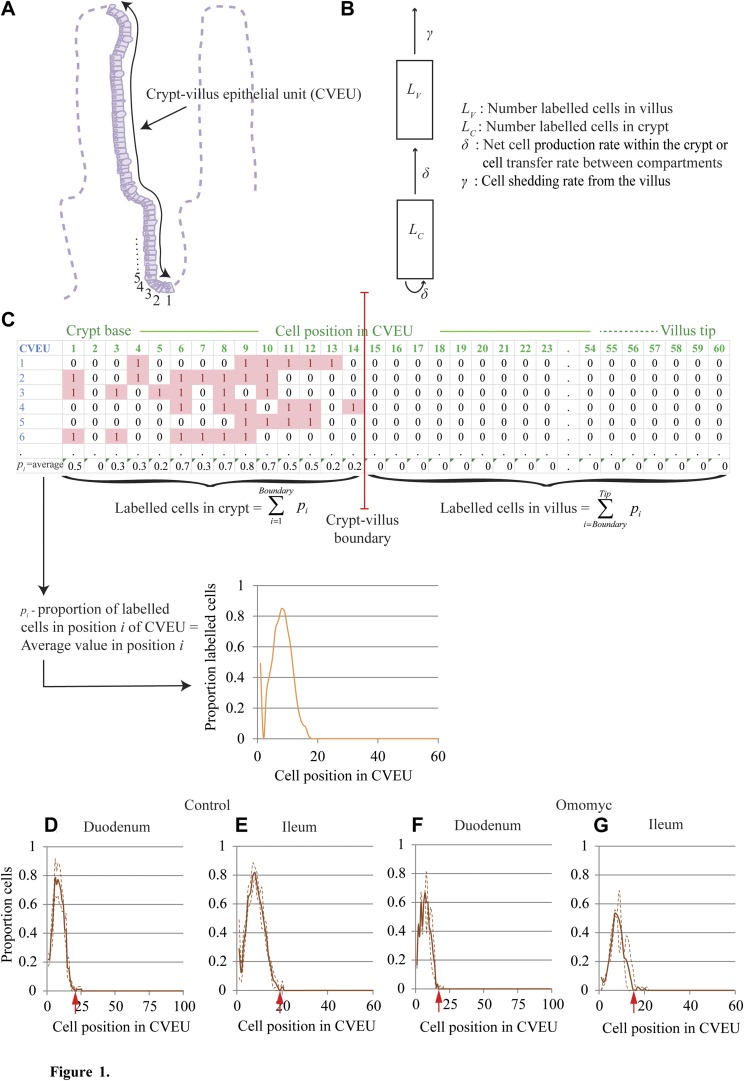
BrdU pulse labeling of the CVEU. *A*) The CVEU, with numbers indicating the location of the first few cell positions. *B*) The 2-compartment model developed to quantify the temporal dynamics of BrdU labeling along the CVEU. C) Representative count data for 6 CVEU counts; at least 30 CVEUs were counted per sample. The proportion of labeled cells at each position of the CVEU was estimated as the average of the scores, 1 or 0, assigned to each position. The crypt–villus boundary was estimated as the lowest position in the upper part of the crypt at which the proportion of BrdU-labeled cells 2 h after injection is smaller than 0.01. The number of labeled cells in the crypt and on the villus are estimated by summing all labeled cell proportions up to and beyond, respectively, the crypt–villus boundary. *D*–*G*) Proportion of BrdU-labeled cells 2 h after BrdU injection in the duodenum and ileum of control (*D*, *E*) and Omomyc (*F*, *G*) mice. Continuous lines show mean values and discontinuous lines se intervals estimated from 3–5 animals. Red arrows: crypt–villus boundary. The length of each horizontal axis reflects the average measured length (in cell number) of the CVEU in each tissue and is based on the results presented in Table 1.

We modeled the propagation of BrdU labeling across these 2 compartments after a pulse of BrdU (**Fig. 1*A, B***). After BrdU injection, at the start of the experimental period, labeled cells were not homogenously distributed within the crypt; they were more abundant in intermediate positions and decreased in number toward the mouth and base of the crypt; hence, the initial transfer of labeled cells from the crypt to the villus compartment was negligible. This cell distribution changed as BrdU-labeled cells proliferated, differentiated, and migrated so that nonproliferative BrdU-labeled cells were generated within the crypt, the distribution of the label became more homogenous, and the transfer of labeled cells to the villus started. On the other hand, shedding of labeled cells did not start until labeled cells reached the tip of the villus.

We assume that, for our experimental conditions, the size of the crypt compartment, *N*_C_, remains fixed at a constant value over time, and hence, cell proliferation within the crypt is compensated for by the transfer of cells to the villus and the rate of cell proliferation is equal to the rate of cell transfer between compartments (see Fig. 1).

For simplicity, we assume that migration of labeled cells from the crypt to the villus is initiated when the number of labeled cells within the crypt reaches a threshold value, *L*_C_*. Similarly, cell shedding from the villus is initiated when the number of labeled cells in the villus reaches a second threshold value, *L*_V_*. Other assumptions are that 2 h after administration, BrdU is no longer available for uptake, and so, BrdU-labeled cells are generated only by the division of previously labeled cells. Moreover, we restricted our attention only to the period of time before the cell BrdU content has been diluted below the detection limit. With these assumptions, the temporal dynamics of our model satisfy the ordinary differential equations in Eq. 1:
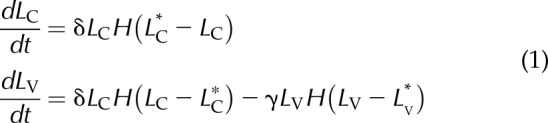
where *L*_C_ and *L*_V_ represent the number of BrdU-labeled cells in the crypt and villus compartments, respectively, at time *t;* δ (h^−1^) represents the specific cell proliferation rate within the crypt and is equal to the specific rate of cell transfer between compartments. By cell proliferation we refer to the net difference between cell proliferation and cell death; γ (h^−1^) is the rate of cell shedding; and 



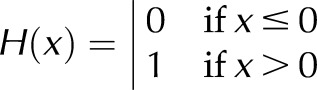
is the Heaviside step function.

For a label that progresses from the crypt to the villus until labeled cells are shed from the tip of the villus, such that γ > 0 and *L*_V_ > *L*_V_*, and imposing initial conditions *L*_C_ (*t*_0_) = *L*_C0_ and *L*_V_ (*t*_0_) = *L*_V0_, the explicit solutions of Eq. 1 take the form of Eq. 2:
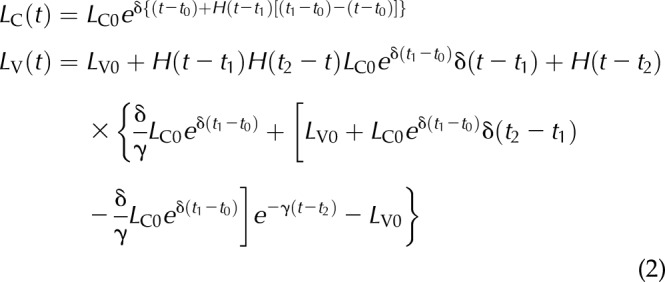
where *t*_1_ and *t*_2_ are such that *t*_2_ ≥ *t*_1_ ≥ *t*_0_ ≥ 0; they denote the times at which the number of labeled cells within the crypt and on the villus reach the threshold values *L*_C_* and *L*_V_*, respectively.

If we restrict attention to the time before shedding of labeled cells starts (*i.e.,* *t* < *t*_2_), then *L*_V_ ≤ *L*_V_* and Eq. 2 reduces to Eq. 3:
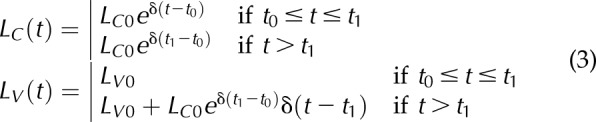
Eqs. 2 and 3 can be rearranged in terms of *L*_C_* and *L*_V_*, by using the following expressions (Eq. 4):
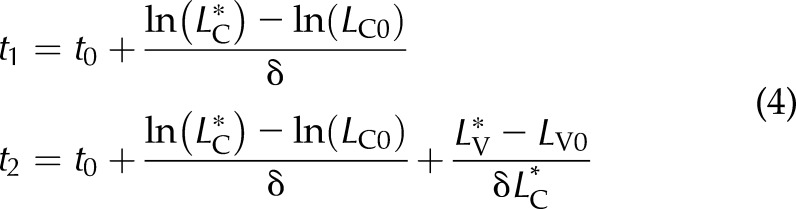
which are obtained by setting *t* = *t*_1_ and *t* = *t*_2_ in the expression for *L*_C_ and *L*_V_ of Eq. 2, respectively.

##### Parameter estimation

Our data were collected before the BrdU-labeled front reached the villus tip and shedding of labeled cells started, and we therefore fitted Eq. 3 to the observed counts of labeled cells in each compartment: crypt and villus. The observed numbers of BrdU-labeled cells in the crypt and villus compartments at each sampling time were calculated as the sums of the proportions of BrdU-labeled cells at all cell positions below and above the crypt–villus boundary, respectively (Fig. 1). The crypt–villus boundary which, as explained above, is equivalent to the size of the crypt compartment, *N*_C_, was estimated as the lowest position in the CVEU at which the proportion of BrdU-labeled cells 2 h after BrdU injection is less than 0.01 (**Table 1**). We demonstrate below and in Supplemental Fig. 2 that the crypt compartment, defined in this way, contains all proliferative cells of the CVEU.

**TABLE 1.** Number of cells and population kinetic parameters in the CVEU

	Control		Omomyc		Within 0–10 h after AraC^*a*^		Beyond 10 h after AraC^*b*^	
Description	Duodenum	Ileum	Duodenum	Ileum	Duodenum	Ileum	Duodenum	Ileum
CVEU length (cells)	99.7 ± 8.30	61.5 ± 7.18	98.8 ± 7.32	52.1 ± 7.36	93.4 ± 7.16	64.7 ± 10.6	101 ± 11.9	59.2 ± 9.25
Cell density in labeled strips (cells/10 µm)	1.25 ± 0.246	1.00 ± 0.129	1.01 ± 0.141	0.865 ± 0.112	0.848 ± 0.0841	0.917 ± 0.102	1.12 ± 0.192	0.778 ± 0.165
Labeled cells 2 h after BrdU (*n)*	8.2 ± 0.4	7.4 ± 0.4	6.1 ± 0.4	4.3 ± 0.5	0	0	8 ± 0.8	6.8 ± 0.3
Crypt–villus boundary; crypt compartment size (*n* cells): *N*_C_^*c*^	20 ± 2.0	19 ± 1.8	18 ± 0.8	16 ± 0.7	20^*d*^	20^*d*^	20 ± 1.0	20 ± 2
Specific cell proliferation rate or cell transfer rate to villus: δ (h^−1^) in Eq. 1	0.0760 ± 0.0147	0.0544 ± 0.00644	0.0547 ± 0.00665	0.0509 ± 0.00427	0.00891 ± 0.0128^*e*^	0.00136 ± 0.00698^*e*^	0.0864 ± 0.0144	0.0466 ± 0.00901
Crypt cell production rate or cell migration velocity at crypt–villus boundary: *V*_C-V_ (cells/h)^*f*^	1.51 ± 0.325	1.03 ± 0.156	0.983 ± 0.126	0.813 ± 0.0768	0.174 ± 0.253^*e*^	0.0279 ± 0.154^*e*^	1.73 ± 0.300	0.934 ± 0.204
Migration velocity of labeled front along villus: *V*_LF_ (µm/h) in Eq. 6	9.00 ± 0.465	5.96 ± 0.379	7.24 ± 0.190	5.29 ± 0.235	−0.798 ± 0.942^*e*^	0.0765 ± 0.477^*e*^	8.68 ± 0.655	6.09 ± 1.15

Average number of cells and standard deviation of 3–6 samples from different mice and estimates and se of fitted parameters.

a Estimated with data obtained within the first 10 h after AraC injection.

b Estimated with data obtained after the first 10 h after AraC injection.

c Estimated as the position at which the fraction of BrdU labeled cells, 2 h after injection, takes values smaller than 0.01.

d Assumed from BrdU data observed after the first 10 h after AraC injection.

e Not significantly different from 0.

f Estimated as 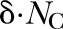.

As explained in Results (Supplemental Fig. 1), experimental “time 0” was set to 2 h after a single injection of a thymine analog molecule. In control and Omomyc mice, both *t*_0_ (initial time) and *L*_V0_ (initial number of labeled cells on the villus) were given fixed values: *t*_0_ = *L*_V0_ = 0. For the dataset collected within the first 0–10 h after Ara-C injection, the prescribed value of *t*_0_ was 15 h, which corresponded to the first sampling time after IdU label activation; for the dataset collected beyond 10 h after Ara-C injection, *t*_0_ was fixed to 25 h, which accounted for the extra 10 h of arrested proliferation. For both datasets derived from Ara-C-treated mice, we assumed that the value of *t*_1_ (the time at which cell transfer from crypt to villus starts) was equal to *t*_0_, reflecting the fact that the IdU-labeled front was already on the villi at *t*_0_. The remaining parameters in control and Omomyc mice, *t*_1_, δ (specific proliferation rate), and *L*_C0_ (initial number of labeled cells in crypt), and in Ara-C-treated mice, δ, *L*_C0_ and *L*_V0_, were estimated by fitting Eq. 3 to the number of labeled cells in each compartment, using the nonlinear regression procedure of SAS 9.3 (SAS, Cary NC, USA). The function log(*L* + 1), where *L* is the number of labeled cells, was used for variance homogenization purposes in the fitting process. Supplemental Table 1 shows the fitting results for each experimental dataset.

Furthermore, we estimated the net cell production rate, in our 1-dimensional CVEU crypt compartment, by the product δ *· N*_C_, where δ denotes the specific cell proliferation rate in our 2-compartment model, and *N*_C_ is the total number of cells, both proliferative and nonproliferative, within the crypt. In that the CVEU we have defined is a 1-dimensional column of cells, the size of the crypt compartment, *N*_C_, is equal to the position of the crypt–villus boundary.

The velocity (cell lengths/h or cells/h) induced by mitotic pressure of a cell located at any position *x* within the crypt, *V_x_*, was calculated as (Eq. 5):
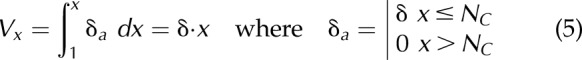
Thus, the velocity of a cell at the crypt–villus boundary is determined by *V*_C_*_-_*_V_ = δ *· N*_C_, which is also the cell migration velocity on the villus related to crypt cell proliferation and is equivalent to the crypt cell production rate defined above. We made the simplifying assumption that the specific cell proliferation rate is constant across cell positions in the crypt and equal to the average rate estimated for the entire crypt; however, the cell cycle duration seems to vary according to position, with longer cycles reported at the base of the crypt than in the transit amplifying compartment (2). Thus, the assumption of constant proliferation rate across the crypt may increase the error of estimation of the cell velocity at positions below the crypt–villus boundary.

We also estimated the time necessary for a cell to migrate from the base of the crypt to the top of the villus as the sum of the migration time within the crypt and the time to migrate across the villus. The time required for a cell to migrate from crypt base to the crypt–villus boundary can be calculated as 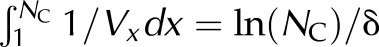, where *V_x_* = δ · *x* is the velocity (cell lengths/h) of a cell at position *x* in the crypt, as described in Eq. 5 and under the assumptions explained above. Assuming that cell migration on the villus is driven by proliferation, the time required for a cell to migrate from the crypt–villus boundary to the tip of the villus results from dividing the number of cells on the villus by the cell velocity at the villus–crypt boundary, *V*_C_*_-_*_V_ = δ *· N*_C_, described above.

##### Evaluating the assumption that the defined crypt compartment contains all proliferative cells of the CVEU

The estimation of the location of the interface between the crypt and villus compartments is based on the assumption that all proliferative cells are located below a selected position which is the lowest position of the CVEU at which the proportion of labeled cells 2 h after BrdU injection is smaller than 0.01. Failure of this assumption would result in erroneous estimation of the absolute cell production in the crypt compartment per hour, or cell velocity at the crypt–villus boundary, estimated by *V*_C_*_-_*_V_ = δ *· N*_C_.

We have analyzed the sensitivity of the model parameters to the position of the crypt–villus boundary. To do that, we selected several positions for the crypt–villus boundary above and below our selected criterion using the dataset generated in the duodenum of control mice and ileum of the Omomyc mice. The model was fitted to the observed number of labeled cells in the crypt and villus compartment for each tested boundary. Similar absolute cell production rates in the crypt were observed for positions of the boundary equal to, or higher than, the position selected with our criterion. A larger number of cells in the crypt compartment, *N*_C_, resulted in compensatory reductions in the fitted values of the specific cell proliferation rate, δ, (Supplemental Fig. 1), demonstrating that our selected crypt–villus boundary is above practically all proliferative cells in the CVEU. Had proliferative cells been located above the selected boundary, variable absolute cell production rates would have been detected for boundaries above our selected position.

#### Estimating cell velocity along the villus compartment from BrdU labeling experiments

We defined the position of the labeled front as the distance in micrometers from the base of the crypt to the location of the highest BrdU-labeled cell on the CVEU. Figure 4 indicates that in our 1-dimensional CVEU the displacement of the labeled front along the villus takes place at constant velocity. Therefore, the average observed velocity of the labeled front on the villus compartment, *V*_LF_ (μm/h), was estimated by linear regression, assuming (Eq. 6):
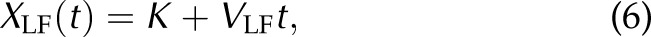
where *X*_LF_ (*t*) is the position of the labeled front at time *t*, and *K* is an arbitrary constant.

#### Statistical inference

The statistical comparison of crypt cell production rates in mouse models and tissues was performed by simulating the posterior probability distribution of the difference in the crypt cell production rate between control mice and other mouse models and between the duodenum and ileum.

**Figure 2.**
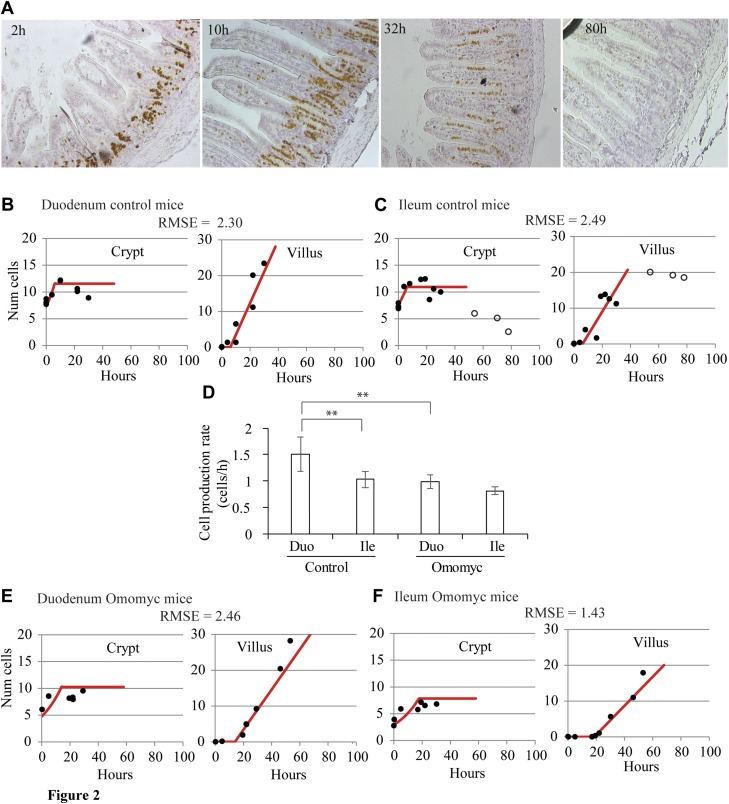
Modeling BrdU labeling in the CVEU, assuming that cell proliferation within the crypt drives migration onto the villus compartment in control and Omomyc mice. *A*) Representative images of BrdU labeling progression along the CVEU of ileum of control mice over 80 h after BrdU injection. *B*, *C*) Experimental observations (circles) and 2-compartment model predictions (lines) of the number of labeled cells over time in the crypt and villus in the duodenum (*B*) and ileum (*C*) of control mice. Filled circles: data points used to fit the model; open circles: data points affected by BrdU cell content dilution resulting from several successive divisions within the crypt; these points were not used for fitting (*C*). *D*) Comparison of net cell production rates in crypts from duodenum and ileum of control and Omomyc mice. Asterisks indicate statistically significant differences. Statistical inference methods and results can be found in Materials and Methods and Supplemental Fig. 3*E*, *F*) Fit of the 2-compartment model (lines) to the number of labeled cells over time (circles) in the crypt and villus of duodenum and ileum of Omomyc mice. The root mean square error (RMSE) between predictions and observations is shown for each model fit. Time 0 is set to 2 h after BrdU administration.

We estimated *P*[*V*_C_*_-_*_V_ (group *i*) > *V*_C_*_-_*_V_ (group *j*) | X ], where X denotes our dataset of observed number of labeled cells across the CVEU over time, and *i* and *j* represent the mouse model tissues in comparison, using Monte Carlo methods. We assumed that *V*_C_*_-_*_V_ was the product of 2 variables δ *· N*_C_ with known posterior probability distributions: δ | X which followed an inverse γ distribution with parameters *k* and θ and *N*_C_ | X, which had a log-normal distribution with parameters µ and σ. The parameters of these distributions are known from the fitted values and associated standard errors of δ and *N*_C_ for each group of mice or tissues (Table 1). The number of simulated values from each distribution was 10,000, which enabled the estimation of the average and error of the simulated random variables with a precision of 3 significant figures. Differences between groups were considered significant when *P*[*V*_C_*_-_*_V_ (group *i*) > *V*_C_*_-_*_V_ (group *j*) | X] > 0.95 (see Fig. 2*F* and Supplemental Fig. 3).

Similarly, we simulated the posterior probability of the ratio between cell velocity at the crypt–villus boundary induced by mitotic pressure, and cell velocity along the villus, *V*_C_*_-_*_V_/*V*_LF_, for each mouse model tissue using Monte Carlo methods. The posterior probability of *V*_C_*_-_*_V_ was simulated as described above; *V*_LF_ | X was assumed to have a normal distribution, with known parameters presented in Table 1 for each tissue. The statistical comparison of the computed ratio among the assayed mouse models and tissues was made by simulating the 95% confidence interval of the difference between their ratios.

## RESULTS

### Development of a 2-compartment model of temporal cell dynamics of BrdU pulse-labeled cells in the CVEU

We defined the CVEU as a 1-dimensional column of cells running from the base of a crypt to the tip of a neighboring villus, as observed on transverse sections of the small intestine (Fig. 1*A*). To investigate cell proliferation and migration along the CVEU, we administered a single pulse of BrdU, tracked the distribution of labeled cells along the CVEU over time, and developed a mathematical model that quantifies the temporal dynamics of BrdU labeling or similar thymine analogs along the CVEU. This model describes the labeling process across 2 compartments: the crypt compartment includes the population of proliferative cells of the CVEU, as well as nonproliferative cells, both populations with numbers we assume to be constant on average; and the villus compartment, which contains nonproliferative cells (Fig. 1*B*). After a single BrdU injection, the model assumes that a proportion of proliferative crypt cells will be labeled. These labeled cells proliferate, differentiate, migrate, and eventually start to be transferred to the villus compartment, where they migrate until they reach the tip of the villus and are shed into the lumen (for full details, see Materials and Methods). Our model assumes that cell transfer between the crypt and villus is exclusively related to cell proliferation. This assumption arises naturally when considering that, in our experimental conditions, the size of the crypt compartment maintains a constant value, *N*_C_, over time. Hence, cell proliferation within the crypt is compensated for by the transfer of cells to the villus, and these processes take place at the same rate. Given that our CVEU is 1-dimensional, *N*_C_ is also the length of the crypt, expressed as the number of cells. By “cell proliferation” or “cell production” we mean the net difference between cell proliferation and cell death. Our model also assumes that BrdU cell content is not yet diluted by successive cell divisions to below the detection limit. To comply with that assumption, the experimental time was limited so that, in practice, all our data were collected before the BrdU-labeled front reached the villus tip. The model then admits an explicit solution, detailed in Eq. 3, which we fitted to the counts of BrdU-labeled cells observed in each compartment.

To collect such counts of labeled cells at several times after a single BrdU injection, we assigned each cell position along the CVEU the value 1, if the cell at that location was labeled with BrdU, or 0, if it was not labeled (Fig. 1*C*). The proportion of BrdU-labeled cells at each position of the CVEU was estimated as the average of the scores, 1 or 0, at that cell position for at least 30 CVEUs per mouse per time point.

We first determined the time needed for cell labeling after a single BrdU injection. Samples collected any time between 30 min and 2 h after BrdU injection in control mice yielded similar BrdU labeling profiles along the ileum CVEU (Supplemental Fig. 1*A*), indicating that 30–45 min after injection, BrdU was not available for uptake and that possible cell proliferation occurring within the 2 h period after BrdU injection did not affect the distribution of labeled cells. We also verified in Omomyc mice, with reduced cell proliferation, that samples collected 2 and 4 h after BrdU injection yielded similar BrdU-labeled proportions (Supplemental Fig. 1*B*). We concluded that 2 h after a single-dose BrdU injection constituted an adequate time 0 in the BrdU labeling process for all mouse models and tissue samples.

Using the profile of labeled cells along the CVEU observed 2 h after BrdU injection, we next estimated the position of the boundary between the crypt and villus compartments. To ensure that all proliferative cells were contained within the crypt compartment, the boundary was estimated as the lowest cell position along the CVEU at which the proportion of BrdU cells observed 2 h after injection was smaller than an arbitrarily chosen small number equal to 0.01 (Fig. 1*D*–*G* and Table 1). We demonstrated in Materials and Methods and Supplemental Fig. 2 that this position is above practically all proliferative cells. As the CVEU is a 1-dimensional column of cells, the position of the crypt–villus boundary coincides with the size of the crypt compartment *N*_C_ or the total number of cells, both proliferative and nonproliferative, within the crypt.

Once the boundary was established, we calculated the number of BrdU-labeled cells in the crypt and villus compartments at each sampling time as the sums of the proportions of BrdU-labeled cells at all cell positions below and above the crypt–villus boundary, respectively (Fig. 1*C*).

Our 2-compartment model was first evaluated on BrdU-labeled CVEUs of ileum and duodenum of healthy mice (**Fig. 2*A*–*C***). Not all data points comply with the required conditions to fit the model. For example, in the ileum of control mice (Fig. 2*C*), we observed that ∼40 h after BrdU injection, the number of BrdU-labeled cells within the crypt started to decrease, probably because of the BrdU cell content dilution during successive divisions. At similar sampling times, the number of BrdU-labeled cells on the villus reached a maximum constant value, which is likely to have been caused by cell shedding from the villus tip. These timings are approximate and specific for the ileum in control mice and vary, depending on the length of the CVEU and the population cell kinetics of the tissue of each strain of mouse. Because the 2-compartment model does not account for label dilution, those data points showing these effects were not used for model fitting (Fig. 2*C*). Estimated parameter values and associated errors of model parameters are given in Supplemental Table 1.

**Figure 3.**
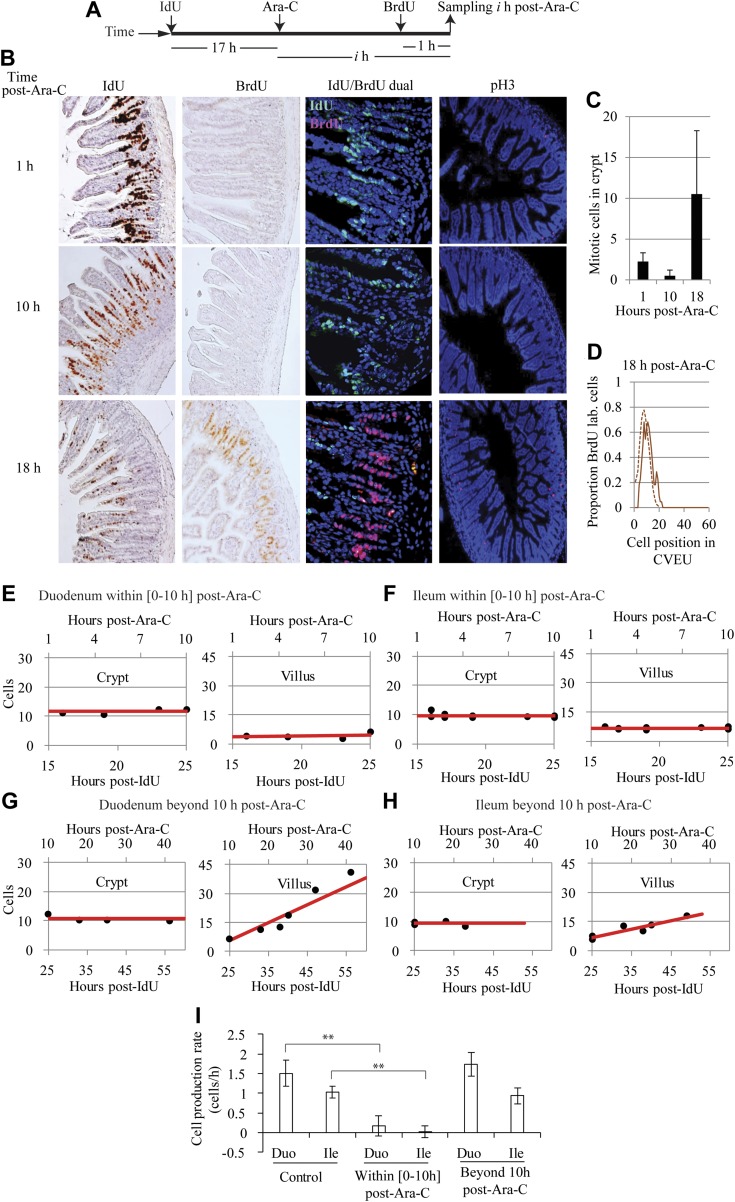
Inhibition of proliferation by treatment with Ara-C. *A*) Experimental strategy for monitoring migration of IdU-labeled cells on the villi and cell proliferation within the crypts after Ara-C administration. *B*) Images of labeled ileum sections at several sampling times illustrating that at the time of Ara-C administration, the IdU-labeled cells had already reached the villi, BrdU uptake was not detected, and mitosis was reduced during the first 10 h after Ara-C administration but recovers at later times. *C*) Number of mitotic events detected by pH3 staining after Ara-C injection. *D*) The proportion of crypt cells incorporating BrdU 18 h after Ara-C injection (continuous line) was similar to that observed in nontreated animals (dashed line). *E*–*H*) Fit of the 2-compartment model (lines) to observed numbers of labeled cells (circles) in the crypt and villus of mice treated with Ara-C. Proliferation within the crypt and cell transfer to the villus were blocked during the first 10 h after Ara-C administration in duodenum (*E*) and ileum (*F*) and resumed in both tissues at later time points (*G*, *H*, respectively). *I*) Comparison of net cell production rates in the crypts of control and Ara-C-treated mice. Asterisks: statistically significant differences. Statistical inference methods and results are in Materials and Methods and Supplemental Fig. 3.

We defined the net cell production rate in the 1-dimensional CVEU crypt compartment, referred to as crypt cell production hereafter, by the product δ *· N*_C_, where δ denotes the specific cell proliferation rate in our mathematical model, and *N*_C_ is the total number of cells, both proliferative and nonproliferative, within the crypt. We found that the crypt cell production rate was ∼1.5 cells/h in the duodenum and significantly lower, about 1 cell/h, in the ileum of healthy mice (Fig. 2*D* and Table 1). In agreement with these results, equivalent rates, estimated with *S*-phase cell labeling techniques, have been reported to decrease in the distal direction of the small intestine, from 1.5 to 1 cell/h (2). Our mathematical model assumes that cell transfer from the crypt to the villus is related exclusively to cell proliferation within the crypt. The plausibility of the fitted parameter values and the agreement between the model and the data (Fig. 3*B*, *C*) indicate that the proposed hypothesis (*i.e.,* cell proliferation forces cell migration from the crypt to the villus), represents a credible explanation for the observed cell dynamics in these compartments.

### Cell proliferation is reduced in the small intestinal epithelium of Omomyc mice

We next studied cell proliferation and migration along the intestinal crypt–villus axis in the *TRE-Omomyc;actin-rtTA* double-transgenic mouse, which is reported to exhibit decreased cell proliferation in multiple tissue sites, including the intestine (29). The reduction in cell proliferation in these mice results from inhibition of the basic helix–loop–helix leucine zipper (bHLHZip) transcription factor Myc, which normally coordinates cellular programs regulating cellular growth, proliferation, tumorigenesis, and apoptosis. Myc inhibition is mediated by a doxycycline-induced mutant bHLHZip protein, omomyc, which sequesters and inhibits Myc, in a dominant-negative fashion (32). Sustained omomyc expression in the small intestines of these mice, which we have referred to throughout as Omomyc mice, results in a reduction in Ki-67 proliferative cell staining in crypts and significantly blunted villi, yet epithelial integrity is maintained and there are no apparent effects on apoptosis or differentiation (29).

Expression of Omomyc protein in intestinal crypts was confirmed by specific antibody staining (Supplemental Fig. 4), adding to previous reports of Omomyc mRNA expression in the intestine (29). The total number of BrdU-labeled cells 2 h after injection was reduced in Omomyc mice, compared with control mice (Table 1), indicating that crypt cell proliferation was reduced when Omomyc expression was induced. To accurately quantify cell proliferation, we fitted the 2-compartment model in Eq. 3 to the number of BrdU-labeled cells in the crypt and villus compartments of Omomyc mice observed over time (Fig. 2*E*, *F*) fitting results are given in Supplemental Table 1. In Omomyc mice, we found the crypt cell production rate to be smaller than in control mice and greater in the duodenum than in the ileum (Table 1). Although differences were close to statistically significance at the 95% confidence level (Supplemental Table 3), only the rate in the duodenum of Omomyc mice was flagged as significantly slower (0.98 cells/h) than that of control mice (1.5 cells/h) at that confidence level (Fig. 2*D* and Table 1). The observed reduction in the crypt proliferation potential makes this mouse model suitable for studying the impact of cell proliferation on cell migration on the villus as detailed below.

### Cell proliferation within the crypt and cell transfer to the villus are temporally halted by Ara-C administration

We next sought to quantify cell dynamics in a system with temporal blockade and resumption of crypt cell proliferation. We assessed the effect of arrested cell proliferation on crypt–villus transfer, using double labeling with 2 distinct thymine analogs, BrdU and IdU, in mice treated with the cytostatic/cytotoxic agent Ara-C. With multiple high doses, Ara-C treatment of rodents is reportedly lethal to most or all proliferative intestinal crypt cells (33–38), whereas at lower doses, proliferative cells within the crypt exhibit a temporary inhibition of DNA synthesis and can resume cycling and division after a matter of hours (39, 40).

**Figure 3*A*** shows our experimental strategy, which involved the administration of IdU 17 h before the injection of a single dose of Ara-C so that the IdU-labeled front had reached the villi by the time the experimental Ara-C treatment period commenced. Samples were then collected over the following 57 h, with BrdU administered 1 h before euthanasia at each sampling time, to monitor any DNA synthesis within the crypt.

From 1 h after delivery of Ara-C, there was no active DNA synthesis in the crypt, as reflected by the absence of BrdU uptake by cells, indicating that Ara-C successfully halted cell proliferation. This effect lasted for at least 10 h (Fig. 3*B*) and was supported by a dramatic reduction in the number of mitotic figures, as detected by anti-phospho-H3 (pH3) staining, during this period (Fig. 3*C*). A few observed residual mitoses were attributable to dividing cells that had already completed the *S* phase at the time of Ara-C administration. Cell proliferation was resumed in samples obtained 18 h after Ara-C treatment, in which the proportion and distribution of cells incorporating BrdU in the crypt was similar to that observed in nontreated animals (Fig. 3*D*) and the number of pH3-stained cells exhibited a concomitant increase (Fig. 3*C*). In a synchronized fashion, displacement of the labeled front along the villus, of both the duodenum and ileum, was detected in samples collected beyond the first 10 h after Ara-C administration (Supplemental Fig. 5).

To investigate the effect of crypt cell proliferation arrest and its recovery on cell transfer to the villus, we analyzed cell dynamics in 2 distinct periods after Ara-C administration: during the first 10 h after Ara-C injection when cell proliferation was arrested; and afterwards, when proliferation was resumed. To do this, we fitted the 2-compartment model in Eq. 3 to the number of BrdU-labeled cells in the crypt and villus compartments observed during these 2 phases (Fig. 3*E*–*H* and Supplemental Table 1). We observed no increase in the number of IdU-labeled cells on the villi of either duodenum or ileum during the crypt cell proliferation arrest period (Fig. 3*E*, *F*). In agreement with this observation, the fitted cell transfer rates to the villus were not significantly different from 0 (Table 1). During the subsequent period of resumed proliferation, the number of IdU-labeled cells on the villi increased over time (Fig. 3*G*, *H*), resulting in cell transfer rates similar to those of control mice (Table 1) and crypt cell production rates not significantly different from those detected in the ileum and duodenum of control mice (Fig. 3*I*). The number of IdU-labeled cells within crypts maintained a relatively constant value during both periods of arrested and resumed cell proliferation (Fig. 3*E*–*H*), a result consistent with this compartment having reached its maximum number of labeled cells by the time Ara-C was injected. In summary, we found that epithelial cell transfer from crypts to villi is halted and resumes in synchrony with crypt cell proliferation.

### Cell proliferation within the crypt is the principal driving force for cell migration along the villus

We next explored the hypothesis that cell proliferation within the crypt drives cell migration along the villus, by comparing the velocity at which cells migrate out of the crypt, due to cell proliferation, with the velocity at which the labeled front migrates along the villus axis in our homoeostatic-, Omomyc-, and Ara-C-treated mouse tissues.

As described in Materials and Methods (Eq. 5), we defined the velocity, *V_x_* (cells/h, meaning average cell lengths/h), caused by mitotic pressure, of a cell located at any position *x* within the crypt by *V_x_* = δ · *x*, with 1 ≤ *x* ≤ *N*_C_, where δ denotes the specific cell proliferation rate in our 2-compartment model, and *N*_C_ is the crypt–villus boundary. Hence, the velocity of a cell at the crypt–villus boundary can be determined by *V*_C_*_-_*_V_ = *δ · N*_C_ which is equivalent to the crypt cell production rate defined above in our 1-dimensional CVEU (Table 1).

**Figure 4.**
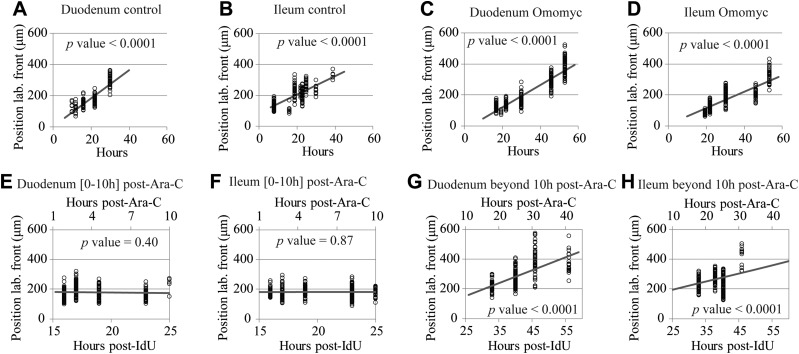
Cell migration velocity along the villus. Observed position of the BrdU-labeled front on the CVEU (circles) and its regression line over time in the duodenum (*A*, *C*, *E*, *G*) and ileum (*B*, *D*, *F*, *H*) of control mice (*A*, *B*) and Omomyc mice (*C*, *D*), 0–10 h after Ara-C treatment (*E*, *F*) and beyond 10 h after Ara-C administration (*G*, *H*).

The average velocity of cells migrating along the villus, *V*_LF_ (μm/h) was estimated by tracking the position of the labeled front, which we measured as the distance in micrometers from the base of the crypt to the location of the highest BrdU-labeled cell on the CVEU, over time, (Fig. 4 and Table 1). Our observations were consistent with the labeled front moving along the villi at a constant velocity (Fig. 4*A–D*), which was estimated by linear regression (Eq. 6), as described in Materials and Methods and Supplemental Table 1. We found that cells migrated faster on the villi of the duodenum than the ileum, and migration was slower in Omomyc mice than in control mice (Table 1). The cell velocity on the villus was not significantly different from 0 during the first 10 h after Ara-C administration (Fig. 4*E*, *F* and Table 1) and fully recovered afterward. These results are consistent with our results regarding the estimated cell velocities at the crypt–villus boundary generated by crypt cell proliferation.

**Figure 5.**
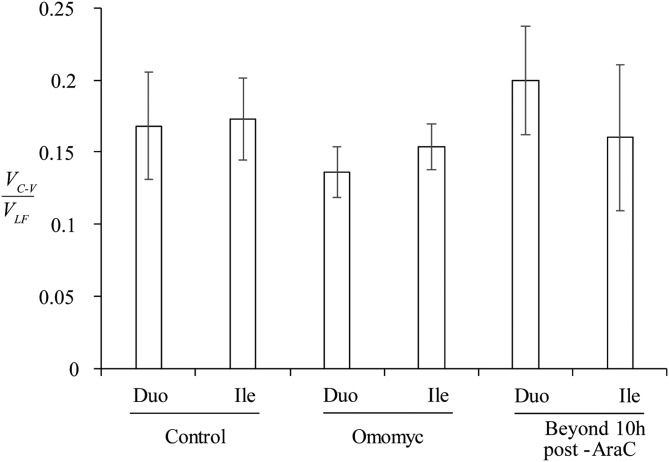
Relationship between cell velocity at the crypt–villus boundary, *V*_C_*_-_*_V_, and cell velocity of the labeled front along the villus, *V*_LF_. The ratio between these velocities was estimated for control and Omomyc mice and mice in which proliferation was resumed following Ara-C treatment. Statistically significant differences were not detected between ratios at 95% confidence level.

We estimated the ratio between *V*_C_*_-_*_V_ and *V*_LF_ in each analyzed case, as described in Materials and Methods, with the exception of samples collected in the first 10 h after Ara-C injection where velocities in both the duodenum and ileum were not significantly different from 0 (Table 1). **Figure 5** shows that the ratio of these velocities was similar across all analyzed tissues, indicating a tight coupling between crypt proliferation rates and villus migration rates. Because of the different distance units between the parameters *V*_C_*_-_*_V_ (cells/h), and *V*_LF_ (μm/h), the constant ratio between *V*_C_*_-_*_V_ and *V*_LF_ observed across the tissues is an estimation of the cell density (cells/μm) of the CVEU. We sought to explore cell density across the CVEU in greater detail by dividing the number of cells in the labeled strip by its length in micrometers measured at different time points and therefore for different strip lengths. Our results indicated that cell density seems to be independent of spatial position across the CVEU (Supplemental Fig. 6) with values of about 1 cell/10 µm, relatively large measurement error and without exhibiting significant differences among the analyzed tissues (Supplemental Fig. 6 and Table 1). The observed constant cell density in the continuous epithelial barrier indicates that cell length is constant along the CVEU. This result, which we confirmed by visual inspection, differs from previously reported observations (4).

The constant ratio between cell velocity at the crypt–villus boundary and that of the labeled front on the villus indicated that reduced/increased rates of net crypt cell production in the duodenum and ileum were met with proportional reductions/increases in cell migration velocity on the villus in all studied mouse models. These results support the hypothesis that cell proliferation within the crypt is the principal force driving cell migration along the villus.

## DISCUSSION

Using a combination of *in vivo* labeled cell tracking and mathematical modeling, we have described a method for precise quantification of cell proliferation and cell migration in the intestinal epithelium. We use a 2-compartment model to quantify epithelial cell dynamics in mouse models with differing cell proliferation potential. We show that in an experimental model of proliferation arrest, migration is halted until proliferation resumes, and that villus cell migration velocity and crypt cell production rate are tightly coupled. Altogether, these results strongly suggest that mitotic pressure is the primary force driving cell migration along the crypt–villus axis. This hypothesis is supported by other experimental analyses: increasing crypt proliferation (using carcinogens, steroids, bacteria, or genetic alteration) is met by an increase in migration (41–46), while reducing mitotic rates, by inhibiting neural activity, results in reduced epithelial migration (47). However, given other studies that report an apparent uncoupling of proliferation and migration (11–18, 30), we cannot rule out the possibility that some additional mechanisms, such as active migration and/or villus contraction and expansion (20–25), may, in some cases, affect the tight coupling between cell proliferation and migration along the crypt–villus axis.

In the Ara-C-treated intestines, we did not detect BrdU uptake and significant cell migration on the villus in our samples until 18 h after Ara-C treatment (Fig. 2*B*–*D* and Supplemental Fig. 4), indicating that cell proliferation resumed sometime between 10 and 18 h after Ara-C treatment. If we assume that cell proliferation and migration restarted at the rates observed in the healthy ileum, 0.83 cells/h and 6.09 µm/h, respectively (Table 1), a period of 9.2 h would be required for the BrdU-labeled front to migrate from the average position at which it was held during the first 10 h after Ara-C (178 µm), to the position reached 18 h after treatment once proliferation and migration resumed (234 µm; Fig. 2*F*). This calculation narrows down the prediction for resumption of proliferation and migration to ∼10 h after Ara-C and is consistent with previous studies in rats, where intestinal crypt cell mitoses were shown to resume around 10–12 h after Ara-C, before returning to homeostatic levels within 16–24 h after Ara-C treatment (48).

By using cell velocity at the crypt–villus boundary, which we have demonstrated is coincidental with cell velocity on the villus, we estimate that the time required for a cell to migrate from the crypt–villus boundary to the tip of the villus in healthy mice is 49 h in the ileum (where villus length is equal to 42.5 cells; Table 1), and 60 h in the duodenum (where the villus length is 80 cells; Table 1). This compares to ∼43 h, for both ileum and duodenum, calculated by Hagemann and colleagues (49), and 36 and 38 h, respectively, reported in BALB/c mice (16), likely reflecting methodological or mouse strain variation or both. To calculate the total time for a cell to migrate from the base of the crypt to the tip of the villus we need to add the migration time within the crypt, which can be estimated as described in Materials and Methods. Under our assumptions, a cell requires on average 92 h to migrate from the base of the crypt to the villus tip in the duodenum of control mice. In Omomyc mice, where proliferation is reduced, this time increases to 135 h in the duodenum. In the ileum, however, villi were shorter in Omomyc mice than in control animals (52 *vs.* 61.5 cells from crypt base to villus tip) resulting in similar calculated transit times for both mouse modes (97 h in Omomyc *vs.* 95 h in control) despite the decreased crypt production and migration rates seen in Omomyc mice.

Our mathematical model is applicable to any *in vivo* model of intestinal dynamics in which cells have been tracked with a label such as a thymine analog or any label that is transmitted to the progeny in a 2-compartment system with similar cell dynamics. Such methods can be used to interrogate epithelial dynamics in *in vivo* models and *in vitro* culture systems in states of perturbed homeostasis and to detect situations in which proliferation and migration become uncoupled and homeostasis is lost. For example, these methods can be used to determine the effects on proliferation and migration of conditions including disease models, pharmacological treatment, genetic alteration, and altered immune states. This application is of particular interest, given that dysregulation or uncoupling of epithelial proliferation and migration is a feature of various human intestinal disorders. Increased cell proliferation is one of the early indicators of cancer development in the intestine (50), yet precancerous cells migrate slowly and have increased residence times in the intestine (51–53). In patients with celiac disease, hyperproliferation in the crypts (54) and a lack of compensation on the villi lead to an anomalous relationship between villi and crypts and a denuded epithelium (54). In this work, we sought to further our understanding of the mechanisms underlying the maintenance of the equilibrium between crypts and villi in the intestinal epithelium and how this balance is lost and may be regained after perturbation. A deeper understanding of how these processes maintain the health of the gastrointestinal tract will help the development of novel preventive strategies for pathologic intestinal conditions such as celiac disease, ulcerative inflammatory processes, and tumorigenesis.

## CONCLUSIONS

In summary, we conclude that cell proliferation within the crypt is the primary force that drives cell migration along the villus. The presented methods can be used to determine the effects on proliferation and migration of conditions including disease, pharmacological treatment, genetic alteration, and altered immune states, and are of particular interest in those intestinal disorders characterized by the uncoupling of cell proliferation and migration.

## Supplementary Material

Supplemental Data

## Abbreviations

Ara-C: cytosine arabinoside
BrdU: 5-bromo-2′-deoxyuridine
CVEU: crypt–villus epithelial unit
DAB: 3,3′-diaminobenzidine
H3: histone 3
HRP: horseradish peroxidase
IdU: 5-iodo-2′-deoxyuridine
pH3: phospho-histone 3
