# Supplementary material for: Cell proliferation within small intestinal crypts is the principal driving force for cell migration on villi
Source: FASEB J. 2016 Oct 20;31(2):636–49. doi: 10.1096/fj.201601002 (PMC5241155; doi:10.1096/fj.201601002)
Supplement: Supplemental Data [file supp_31_2_636__index.html]

Cell proliferation within small intestinal crypts is the principal driving force for cell migration on villi — Supplemental Data 

# Cell proliferation within small intestinal crypts is the principal driving force for cell migration on villi

## Supplemental Data

- Supplemental Data
- Supplemental Data
- Supplemental Data
- Supplemental Data
