# Supplementary figures and images for: Cell proliferation within small intestinal crypts is the principal driving force for cell migration on villi
Source: FASEB J. 2016 Oct 20;31(2):636–49. doi: 10.1096/fj.201601002 (PMC5241155; doi:10.1096/fj.201601002)

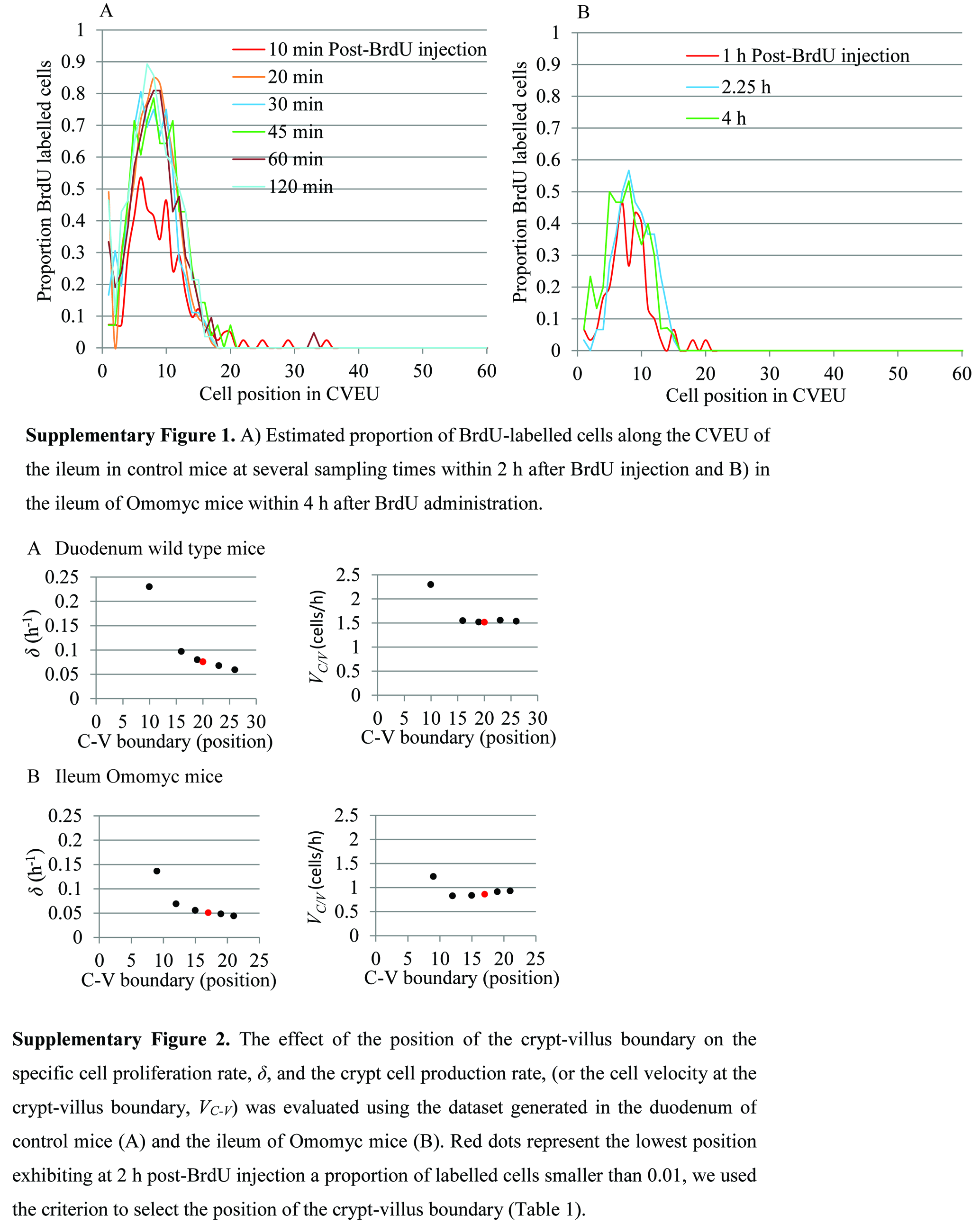

Supplement: Supplemental Data [file supp_fj.201601002_Supplemental_Figures1.tif]

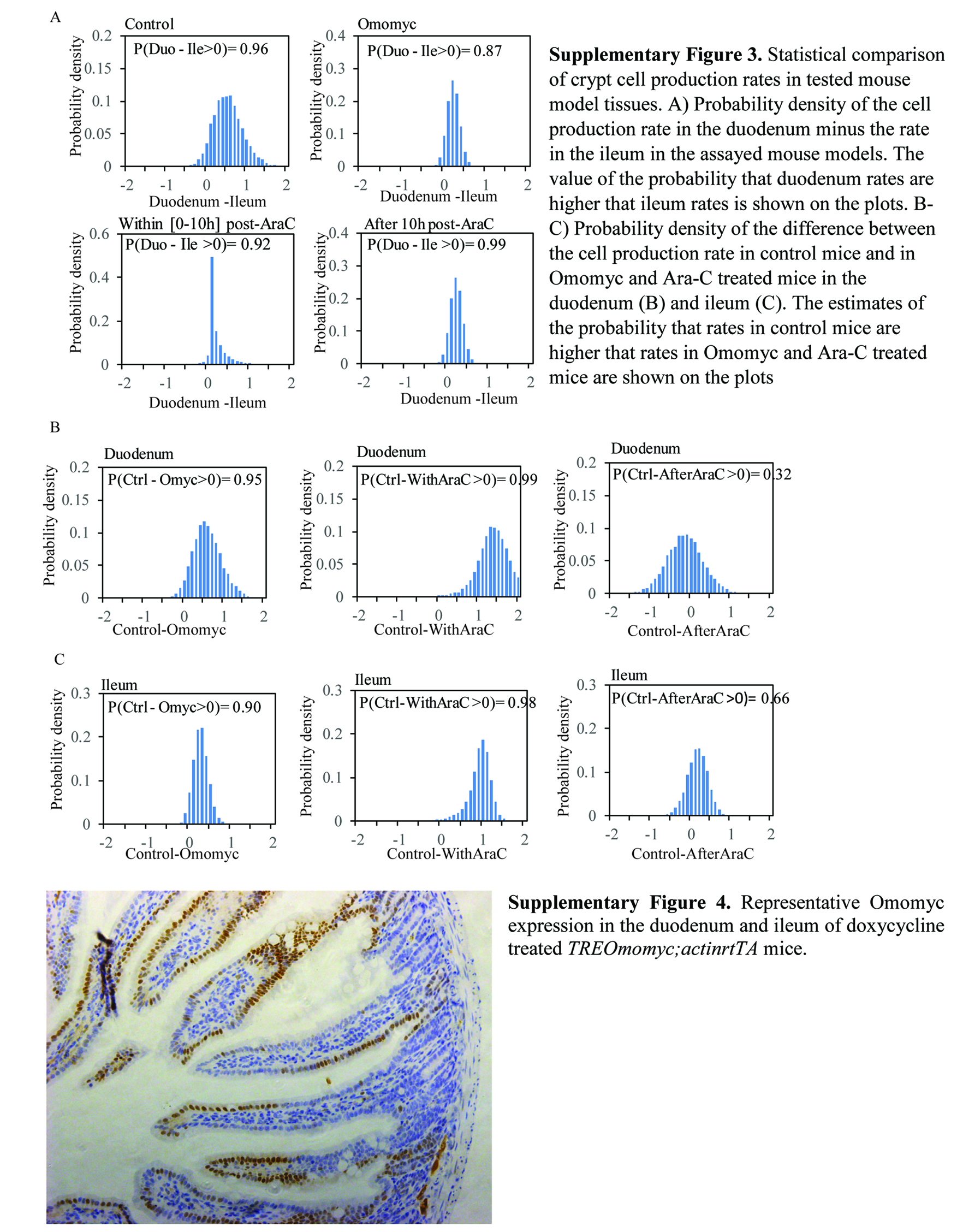

Supplement: Supplemental Data [file supp_fj.201601002_Supplemental_Figures2.tif]

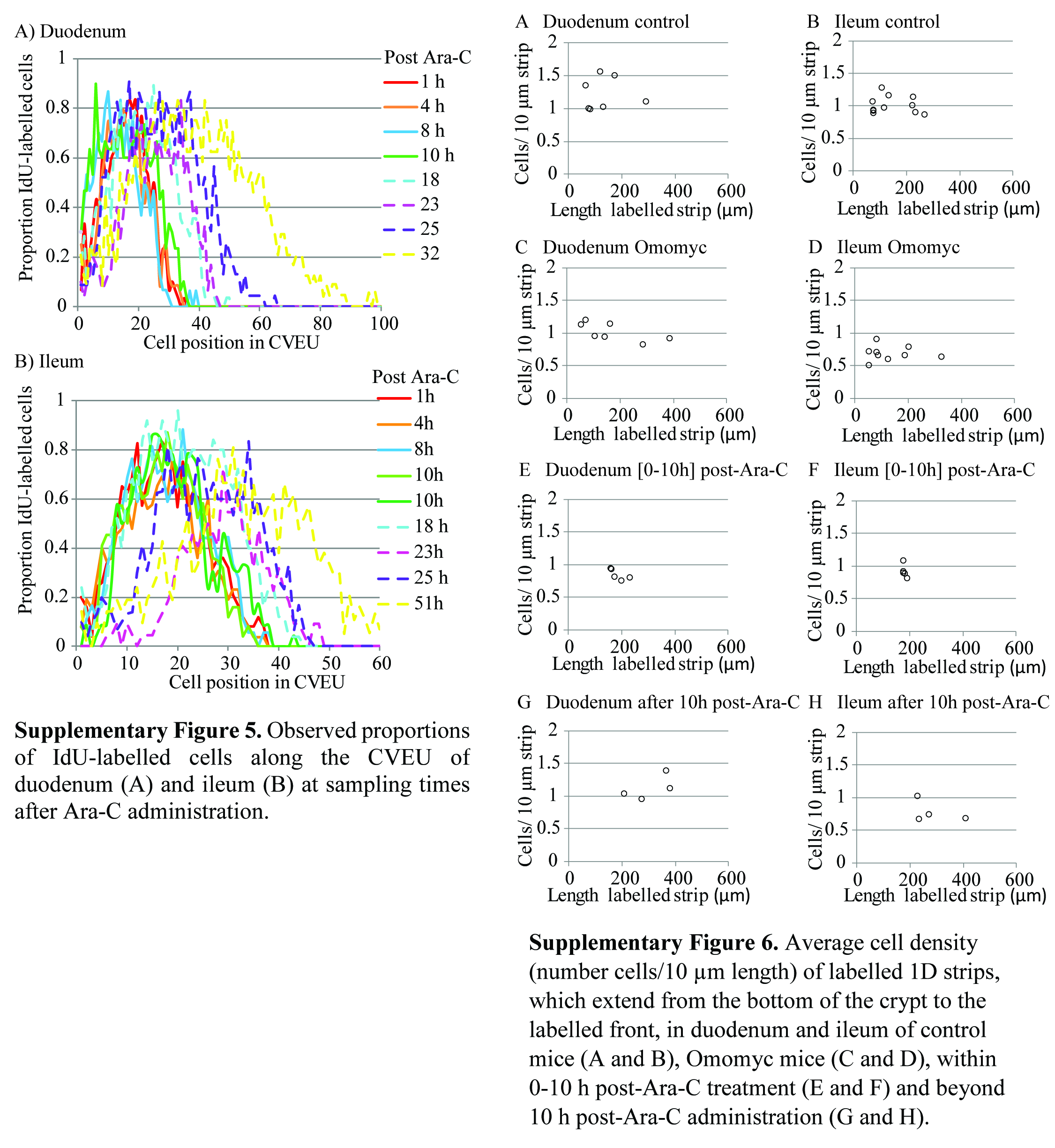

Supplement: Supplemental Data [file supp_fj.201601002_Supplemental_Figures3.tif]
