# Supplementary material for: Cell proliferation within small intestinal crypts is the principal driving force for cell migration on villi
Source: FASEB J. 2016 Oct 20;31(2):636–49. doi: 10.1096/fj.201601002 (PMC5241155; doi:10.1096/fj.201601002)
Supplement: Supplemental Data [file supp_fj.201601002_Supplemental_Table1.pdf]

Supplemmentary Table 1. Model parameters description, estimates and standard error

Parameter (units)	Equation	Description	Control Duodenum	Control Ileum	Omomyc Duodenum	Omomyc Ileum	[3]Within [0-10h] post-AraC Duodenum	[3]Within [0-10h] post-AraC Ileum	[4]Beyond 10h post-AraC Duodenum	[4]Beyond 10h post-AraC Ileum
t_0 (h)	S3	Initial time (h)	[1]0	[1]0	[1]0	[1]0	[1]15	[1]15	[1]25	[1]25
L_{C0} (num cells)	S3	Number of labelled cells in the crypt at t_0	7.32 ± 1.30	7.64 ± 0.947	4.77 ± 0.763	3.09 ± 0.338	11.6 ± 1.29	9.58 ± 0.302	10.71 ± 1.037	9.25 ± 0.669
L_{V0} (num cells)	S3	Number of labelled cells on the villus at t_0	[1]0	[1]0	[1]0	[1]0	3.58 ± 0.81	6.35 ± 1.36	5.60 ± 1.12	6.80 ± 0.689
δ (h^{-1})	S3	Crypt specific cell proliferation rate or specific cell transfer rate from crypt to villus	0.0760 ± 0.0147	0.0544 ± 0.00644	0.0547 ± 0.00665	0.0509 ± 0.00427	[2]0.00891 ± 0.0128	[2]0.00136 ± 0.00698	0.0864 ± 0.0144	0.0466 ± 0.00901
t_I (h)	S3	Starting time of labelled cell migration from crypt to villus	6.00 ± 1.52	7.08 ± 2.25	14.0 ± 1.43	18.6 ± 0.476	[1]15	[1]15	[1]25	[1]25
V_{LF} (µm/h)	S6	Velocity of labelled front on villus	9.00 ± 0.465	5.96 ± 0.379	7.24 ± 0.190	5.29 ± 0.235	[2]-0.798 ± 0.942	[2]0.0765 ± 0.477	8.68 ± 0.655	6.09 ± 1.15
K (µm)	S6	Arbitrary constant	3.28 ± 9.86	86.3 ± 7.54	-25.3 ± 7.21	8.41 ± 8.04	193 ± 17.6	179 ± 9.83	-66.7 ± 27.2	19.9 ± 44.0

[1] Fixed value.

[2] Not significantly different from 0

Time 0 set to time of label activation (2h post-label injection)
